# Palmoplantar Psoriasis Successfully Treated With Risankizumab

**DOI:** 10.7759/cureus.17434

**Published:** 2021-08-25

**Authors:** Abdulmajeed M Alajlan, Tala A Qadoumi

**Affiliations:** 1 Dermatology, King Saud University Medical City, Riyadh, SAU

**Keywords:** psoriasis treatment, biologic agents, "psoriasis, palmoplantar psoriasis, health related-quality of life, risankizumab

## Abstract

Palmoplantar psoriasis is a variant of psoriasis that affects the palms and soles. Despite the small body surface area affected, palmoplantar psoriasis can have significant implications on a patient’s mental health, justifying the urgency in treating this condition. Palmoplantar psoriasis is also known to be challenging to treat. In this case report, we present a male who presented with a 15-year history of psoriasis with significant palmoplantar involvement, managed with topical and systemic therapies, achieving a minimal response. After trying other therapies including acitretin and adalimumab, we eventually started the patient on risankizumab, an anti-IL-23 antibody. Following the fourth dose of risankizumab, the patient’s palmoplantar lesions completely resolved. We further discuss why risankizumab may be considered a treatment option in resistant palmoplantar psoriasis cases.

## Introduction

Palmoplantar psoriasis is reported to have a higher impact on health-related quality of life than moderate to severe plaque psoriasis [1]. This is due to a patient’s inability to carry out simple greeting gestures such as a handshake, leading to social isolation. Therefore, the urgency of its treatment should be taken into consideration. Unfortunately, palmoplantar psoriasis is also frequently resistant to treatment, making it quite a therapeutic challenge [2]. Biologic therapies, such as secukinumab, an anti-interleukin (IL) 17A monoclonal antibody, have been highly effective in the management of resistant palmoplantar psoriasis, as shown in the 2PRECISE randomized controlled trial [3] and GESTURE trial [4]. Risankizumab, another biologic therapy, is a humanized immunoglobulin (Ig) G1 monoclonal antibody that targets the p19 subunit of IL-23 [5]. Risankizumab has been FDA approved since 2019 for the treatment of moderate to severe plaque psoriasis in adult patients, who are candidates for systemic therapy or phototherapy [6]. We present a patient who achieved a complete resolution of palmoplantar psoriasis following four doses of risankizumab, after failing or reaching suboptimal results on other systemic therapies. To our knowledge, this is the first case reported of palmoplantar psoriasis completely resolving with risankizumab.

## Case presentation

A 42-year-old Saudi male presented to our clinic in 2015 with multiple asymptomatic scaly lesions involving the palms, ventral aspect of the wrists, soles, elbows, and popliteal fossae bilaterally. The lesions appeared gradually around 15 years prior to his initial presentation. Up until his presentation to our clinic, the patient was using topical medications such as calcipotriene and betamethasone dipropionate ointment for the lesions and systemic therapies such as acitretin 30 mg once daily. He had no history of any infectious process or stressful life events prior to the onset of the lesions. He did not take any medications, including herbal medication prior to the onset of the lesions. He had no significant past medical history or family history of psoriasis. He was also not complaining of any joint pain. 

**Figure 1:**
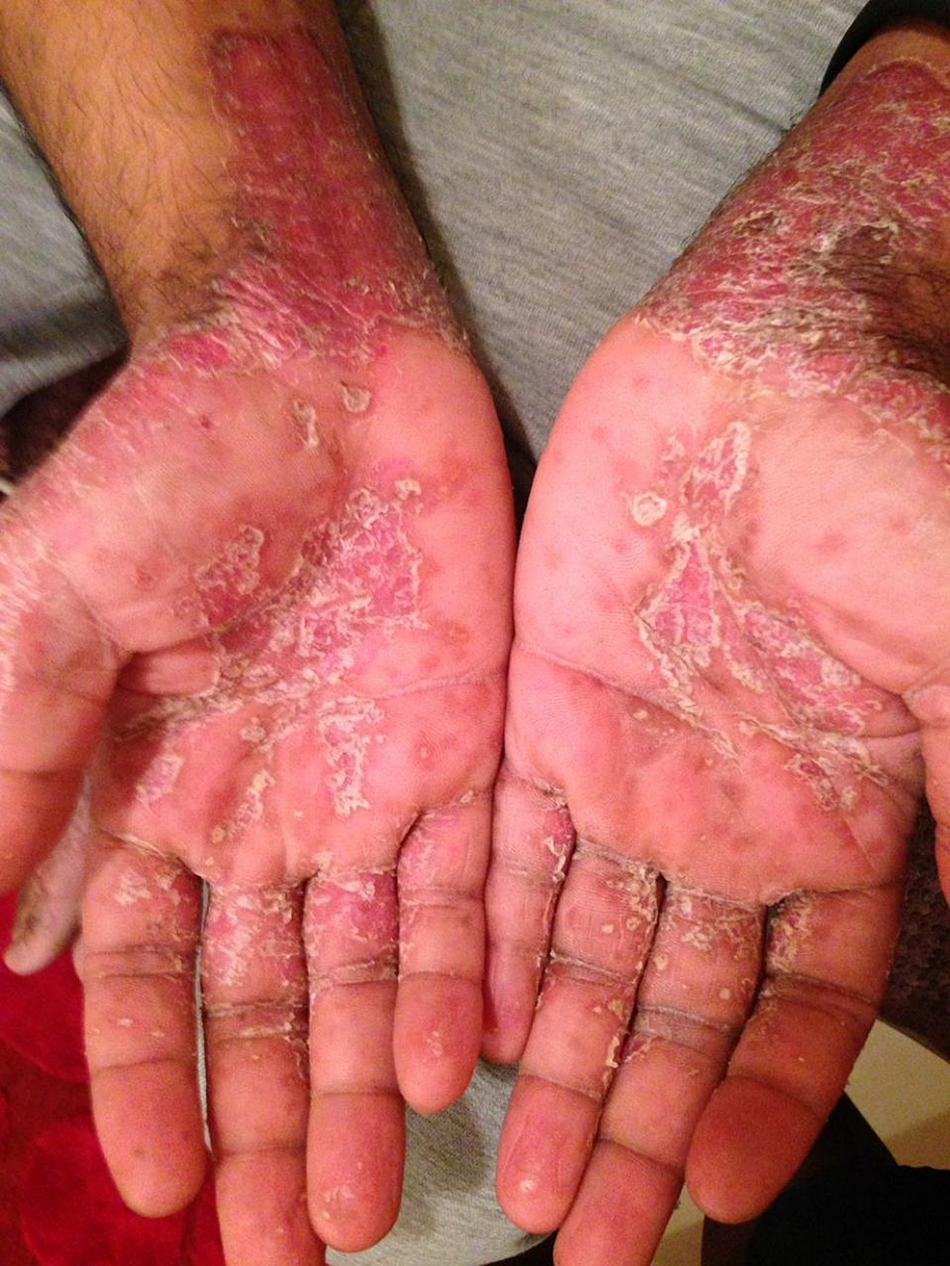
Patient's palms at time of presentation

Physical examination revealed multiple well-demarcated erythematous scaly plaques, some coalescing into larger plaques, involving the palms and ventral aspect of the wrists (Figure 1), soles, popliteal fossae, and elbows bilaterally. There was no nail or mucosal membrane involvement. He was diagnosed with a case of psoriasis with palmoplantar involvement. 

**Figure 2:**
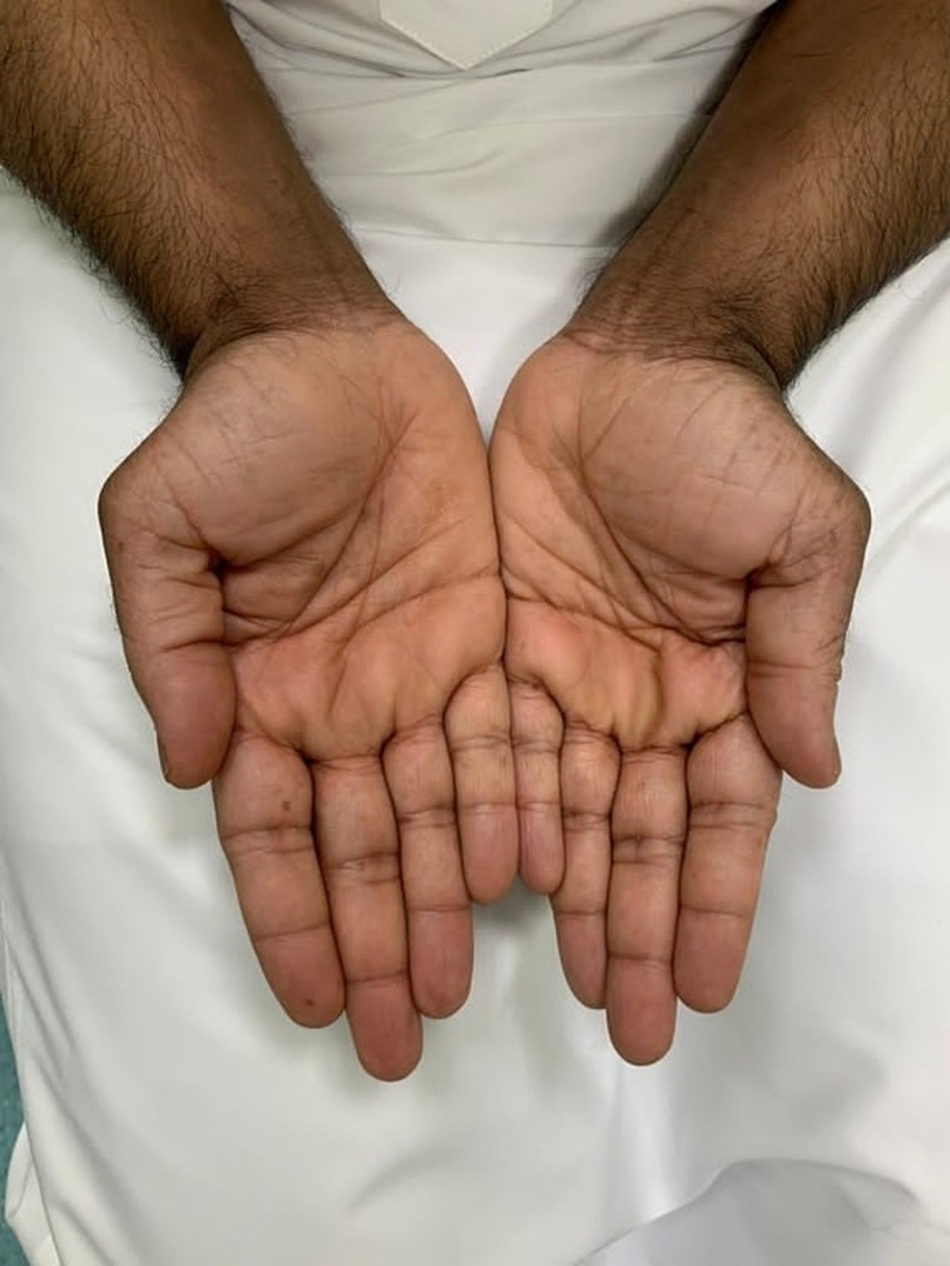
Patient's palms following treatment with risankizumab

He was instructed to continue 30 mg acitretin once daily after relevant laboratory investigations were done. The patient continued taking acitretin for three years but the dose was gradually tapered and discontinued, despite an improvement in psoriatic lesions, due to an abnormal lipid profile. Following the discontinuation of acitretin, the patient relapsed. Therefore, he was started on adalimumab 40 mg subcutaneously every two weeks with minimal improvement after three months of use. Risankizumab was then made available in the hospital pharmacy and the patient was started on it in January of 2020. After the fourth dose of risankizumab, there was a complete resolution of psoriatic lesions, including over the palms (Figure 2) and soles.

## Discussion

Palmoplantar psoriasis is a chronic inflammatory skin disease, accounting for 3%-4% of all psoriasis cases [7]. It is a localized variant of psoriasis, presenting with scaly hyperkeratotic plaques, pustules, or a combination of both, involving the skin of the palms and soles [8]. The body surface area affected is limited, but due to extensive painful fissuring and thickening of the skin surface, lesions may significantly impair daily activities [9]. Palmoplantar psoriasis is a prime example of why body surface area may not always be reflective of the degree of disease severity in psoriasis. The severity of functional impairment should also be taken into consideration when managing these cases [9]. As in psoriasis vulgaris, palmoplantar plaque psoriasis has also been found to be significantly associated with a number of comorbidities such as diabetes mellitus, cardiovascular disease, and mood disorders [10]. Current practices usually employ superpotent topical steroids as first-line topical therapies and acitretin as first-line systemic therapy [11]. Second-line therapies include phototherapy and systemic agents such as methotrexate and cyclosporine [11]. Biologic therapies are typically employed in patients who have failed/cannot tolerate topical therapies and other systemic therapies [11]. Though palmoplantar psoriasis is often resistant to treatment, biologic therapies such as risankizumab may offer promising results.

In order to fully grasp the efficacy of risankizumab in the management of psoriasis, one must fully understand the role that IL-23 plays in the pathogenesis of psoriasis. The IL-23/IL-17 axis is one of the key drivers of psoriasis [12]. Il-23 is produced by antigen-presenting cells. IL-23 binds to the IL-23 receptor on CD4+ T cells resulting in Th17 and Th22 differentiation and the production of many cytokines including IL-17 and IL-22 [13]. These cytokines result in an inflammatory cascade leading to epidermal changes, such as epidermal hyperplasia, seen in psoriatic lesions. IL-23 has two subunits p40 and p19. The p40 subunit is found in both IL-12 and IL-23, while the p19 subunit is unique to IL-23 [14]. This is where risankizumab comes into play, through the inhibition of the p19 subunit of IL-23. Through the inhibition of IL-23, risankizumab will also inhibit IL-17 production. The aforementioned process justifies the use of risankizumab for the treatment of psoriasis, including its variants like palmoplantar psoriasis. Risankizumab also improves patient compliance as it is taken every three months.

## Conclusions

Palmoplantar psoriasis can be frustrating for both physicians and patients. Risankizumab is currently FDA approved for the treatment of moderate to severe plaque psoriasis in adult patients who are candidates for systemic therapy or phototherapy. As seen in our case, risankizumab may offer promising results in the treatment of palmoplantar psoriasis resistant to other systemic therapies.

## References

[1] Chung J, Callis Duffin K, Takeshita J (2014). Palmoplantar psoriasis is associated with greater impairment of health-related quality of life compared with moderate to severe plaque psoriasis. J Am Acad Dermatol.

[2] Rocamora V, Garcías-Ladaria J (2017). Complete response of secukinumab in palmoplantar psoriasis. Dermatol Online J.

[3] Mrowietz U, Bachelez H, Burden AD, Rissler M, Sieder C, Orsenigo R, Chaouche-Teyara K (2019). Secukinumab for moderate-to-severe palmoplantar pustular psoriasis: results of the 2PRECISE study. J Am Acad Dermatol.

[4] Gottlieb AB, Kubanov A, van Doorn M (2020). Sustained efficacy of secukinumab in patients with moderate-to-severe palmoplantar psoriasis: 2·5-year results from GESTURE, a randomized, double-blind, placebo-controlled trial. Br J Dermatol.

[5] Blair HA (2020). Risankizumab: a review in moderate to severe plaque psoriasis. Drugs.

[6] (2021). SKYRIZI (risankizumab-rzaa) injection, for subcutaneous use Initial U.S. https://www.accessdata.fda.gov/drugsatfda_docs/label/2019/761105s000lbl.pdf.

[7] Khandpur S, Singhal V, Sharma VK (2011). Palmoplantar involvement in psoriasis: a clinical study. Indian J Dermatol Venereol Leprol.

[8] Farley E, Masrour S, McKey J, Menter A (2009). Palmoplantar psoriasis: a phenotypical and clinical review with introduction of a new quality-of-life assessment tool. J Am Acad Dermatol.

[9] Engin B, Aşkın Ö, Tüzün Y (2017). Palmoplantar psoriasis. Clin Dermatol.

[10] Greenberg R, Goldsmith T, Zeltser D (2021). Comorbidities in patients with palmoplantar plaque psoriasis. J Am Acad Dermatol.

[11] Miceli A, Schmieder G (2020). Palmoplantar psoriasis. https://www.ncbi.nlm.nih.gov/books/NBK448142/.

[12] Puig L (2017). The role of IL 23 in the treatment of psoriasis. Expert Rev Clin Immunol.

[13] Jeon C, Sekhon S, Yan D, Afifi L, Nakamura M, Bhutani T (2017). Monoclonal antibodies inhibiting IL-12, -23, and -17 for the treatment of psoriasis. Hum Vaccin Immunother.

[14] Haugh IM, Preston AK, Kivelevitch DN, Menter AM (2018). Risankizumab: an anti-IL-23 antibody for the treatment of psoriasis. Drug Des Devel Ther.

